# Individualized positive end-expiratory pressure reduces driving pressure in obese patients during laparoscopic surgery under pneumoperitoneum: a randomized clinical trial

**DOI:** 10.3389/fphys.2024.1383167

**Published:** 2024-04-05

**Authors:** Tiago Batista Xavier, Leonardo Vicente Coelho, Daniel Antonio Lopes Ferreira, José Manuel Cota y Raposeiras, Marcelo Sampaio Duran, Leticia Almeida Silva, Gabriel Casulari da Motta-Ribeiro, Luciana Moisés Camilo, Alysson Roncally Silva Carvalho, Pedro Leme Silva

**Affiliations:** ^1^ Laboratório de Fisiologia da Respiração, Instituto de Biofísica Carlos Chagas Filho, Universidade Federal do Rio de Janeiro, Rio de Janeiro, Brazil; ^2^ Instituto de Educação, Ciência e Tecnologia do Rio de Janeiro, Rio de Janeiro, Brazil; ^3^ Hospital Federal dos Servidores do Estado, Rio de Janeiro, Brazil; ^4^ Laboratório de Investigação Pulmonar, Instituto de Biofísica Carlos Chagas Filho, Universidade Federal do Rio de Janeiro, Rio de Janeiro, Brazil; ^5^ Programa Doutor Empreendedor, Fundação Carlos Chagas Filho de Amparo à Pesquisa do Estado do Rio de Janeiro, Rio de Janeiro, Brazil; ^6^ Instituto D’or de Pesquisa e Ensino, Rio de Janeiro, Brazil; ^7^ Hospital Barra D’Or, Rio de Janeiro, Brazil

**Keywords:** laparoscopy, obesity, pressure, positive end-expiratory, respiratory mechanics

## Abstract

**Introduction:**

During pneumoperitoneum (PNP), airway driving pressure (ΔP_RS_) increases due to the stiffness of the chest wall and cephalic shift of the diaphragm, which favors atelectasis. In addition, depending on the mechanical power (MP) formulas, they may lead to different interpretations.

**Methods:**

Patients >18 years of age with body mass index >35 kg/m^2^ were included in a single-center randomized controlled trial during their admission for bariatric surgery by abdominal laparoscopy. Intra-abdominal pressure was set at 15 mmHg at the pneumoperitoneum time point (PNP). After the recruitment maneuver, the lowest respiratory system elastance (E_RS_) was detected during the positive end-expiratory pressure (PEEP) step-wise decrement. Patients were randomized to the 1) CTRL group: ventilated with PEEP of 5 cmH_2_O and 2) PEEP_IND_ group: ventilated with PEEP value associated with E_RS_ that is 5% higher than its lowest level. Respiratory system mechanics and mean arterial pressure (MAP) were assessed at the PNP, 5 min after randomization (T1), and at the end of the ventilation protocol (T2); arterial blood gas was assessed at PNP and T2. ΔP_RS_ was the primary outcome. Three MP formulas were used: MP_A_, which computes static PEEP × volume, elastic, and resistive components; MP_B_, which computes only the elastic component; and MP_C_, which computes static PEEP × volume, elastic, and resistive components without inspiratory holds.

**Results:**

Twenty-eight patients were assessed for eligibility: eight were not included and 20 patients were randomized and allocated to CTRL and PEEP_IND_ groups (*n* = 10/group). The PEEP_IND_ ventilator strategy reduced ΔP_RS_ when compared with the CTRL group (PEEP_IND_, 13 ± 2 cmH_2_O; CTRL, 22 ± 4 cmH_2_O; *p* < 0.001). Oxygenation improved in the PEEP_IND_ group when compared with the CTRL group (*p* = 0.029), whereas MAP was comparable between the PEEP_IND_ and CTRL groups. At the end of surgery, MP_A_ and MP_B_ were correlated in both the CTRL (rho = 0.71, *p* = 0.019) and PEEP_IND_ (rho = 0.84, *p* = 0.020) groups but showed different bias (CTRL, −1.9 J/min; PEEP_IND_, +10.0 J/min). At the end of the surgery, MP_A_ and MP_C_ were correlated in both the CTRL (rho = 0.71, *p* = 0.019) and PEEP_IND_ (rho = 0.84, *p* = 0.020) groups but showed different bias (CTRL, −1.9 J/min; PEEP_IND_, +10.0 J/min).

**Conclusion:**

Individualized PEEP was associated with a reduction in ΔP_RS_ and an improvement in oxygenation with comparable MAP. The MP, which solely computes the elastic component, better reflected the improvement in ΔP_RS_ observed in the individualized PEEP group.

**Clinical Trial Registration:**

The protocol was registered at the Brazilian Registry of Clinical Trials (U1111-1220-7296).

## Introduction

Millions of laparoscopic procedures are performed globally every year (Boberg et al., 2022), which involve the insufflation of carbon dioxide into the peritoneal cavity to produce a pneumoperitoneum (PNP). PNP can cause dramatic changes in respiratory mechanics due to the stiffness of the chest wall and cephalic shift of the diaphragm, which favors atelectasis (Andersson et al., 2005; Cinnella et al., 2013). Furthermore, the combination of laparoscopic surgery, PNP, and obesity may further increase the negative consequences for the respiratory system (Regli et al., 2019), as observed by an increase in airway driving pressure (ΔP_RS_). In addition, the cardiac output may decrease with a subsequent decrease in the mean arterial pressure (MAP) after an increase in intra-abdominal pressure (IAP) during the PNP procedure (Regli et al., 2019). Different ventilatory strategies have been adopted during the intraoperative period. The recruitment maneuver (RM) followed by the fixed positive end-expiratory pressure (PEEP) level has been associated with an improvement in intraoperative oxygenation in morbidly obese patients and also with hemodynamic instability (Whalen et al., 2006) or functional improvement occurred only after repetitive RM followed by fixed PEEP level titration (Almarakbi et al., 2009; Talab et al., 2009; Edmark et al., 2016). Individualization of the PEEP level after the application of RM has also been done in laparoscopic surgeries in obese patients using pulse oximetry (Ferrando et al., 2018), electrical impedance tomography (EIT) (Nestler et al., 2017), best respiratory system compliance (D Antini et al., 2018; Boesing et al., 2023), or by transpulmonary pressure (Eichler et al., 2018; Mazzinari et al., 2020). However, the use of these variables requires equipment that is not widely available in operating rooms or the introduction of an esophageal balloon, which is not feasible during bariatric surgery under PNP. In addition, the PEEP level has been individualized in laparoscopic surgery in obese patients using several techniques but with minimal changes in real-life ventilator settings in the perioperative period in obese patients. For instance, during the secondary analysis of the international multicenter LAS VEGAS study (Ball et al., 2018), 2,012 obese patients were ventilated with PEEP of 4 cmH_2_O. Recently, RM followed by PEEP titration according to the lowest respiratory system elastance (E_RS_) reduced ΔP_RS_ and cardiovascular performance of the right ventricle in an experimental obesity model and in obese patients (Magder et al., 2021). Nevertheless, the PEEP value associated with minimal E_RS_ may not be feasible during laparoscopic surgery due to hemodynamic instability. Whether ventilating at the PEEP level that corresponds to a 5% increase in E_RS_ according to its minimal value obtained during PEEP titration can maintain the beneficial effects on respiratory system mechanics throughout the surgery without causing hemodynamic instability is not known. Therefore, we hypothesized that a ventilatory strategy based on individualized PEEP (PEEP_IND_; that is, the PEEP level associated with E_RS_ that is 5% higher than the minimal E_RS_) can reduce airway driving pressure compared with PEEP of 5 cmH_2_O and would be hemodynamically feasible.

## Material and methods

### Study design

This is a single-center, randomized controlled trial that evaluated obese patients admitted for bariatric surgery by abdominal laparoscopy at the Hospital Federal dos Servidores do Estado do Rio de Janeiro between September 2018 and December 2019. This study was approved by the Co-substantiated Ethics and Research Committee of the Hospital Federal dos Servidores do Estado do Rio de Janeiro on 14 December 2015 (CAAE: 51623015.9.0000.5252), and written informed consent was obtained from all subjects participating in the trial. The trial was registered prior to patient enrollment at the Brazilian Registry of Clinical Trials (number U1111-1220-7296; http://www.ensaiosclinicos.gov.br/rg/RBR-68y7cz/; principal investigator: Tiago Batista Xavier; date of registration: 28 September 2018). This report follows the CONSORT 2010 Statement: updated guidelines for reporting parallel group randomized trials (Schulz et al., 2010).

### Patient eligibility

The inclusion criteria were patients >18 years of age, with body mass index >35 kg/m^2^, and signed consent provided. The exclusion criteria were chronic obstructive pulmonary disease with forced expiratory volume at first second (FEV_1_) <80% or FEV_1_ divided by forced vital capacity 70% lower after a bronchodilator challenge; heart disease with an ejection fraction <65%; structural chest wall alterations due to obesity such as kyphosis, scoliosis, or hyperlordosis; the absence of consent; and pregnancy.

### Experimental protocol

**FIGURE 1:**
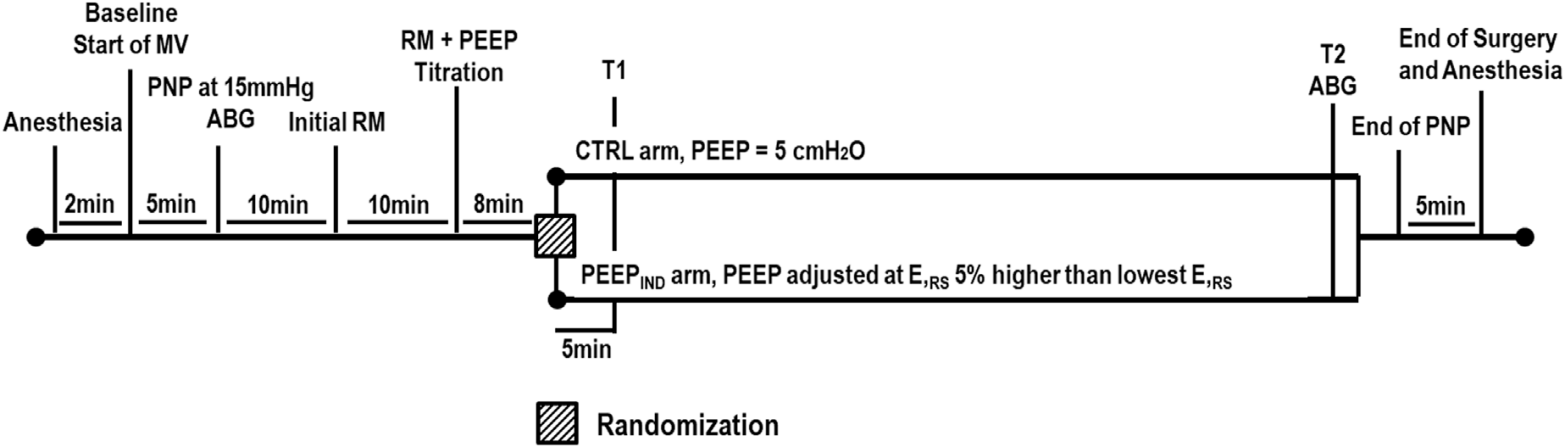
Timeline of the experimental procedures. ABG, arterial blood gases; CTRL, control group ventilated with PEEP of 5 cmH_2_O; E_RS_, respiratory system elastance; MV, mechanical ventilation; PEEP, positive end-expiratory pressure; PEEP_IND_, PEEP adjusted at E_RS_ that is 5% higher than the PEEPminE_RS_; PNP, pneumoperitoneum; RM, recruitment maneuver; T1, at the beginning of surgery; T2, at the end of surgery.

**FIGURE 2:**
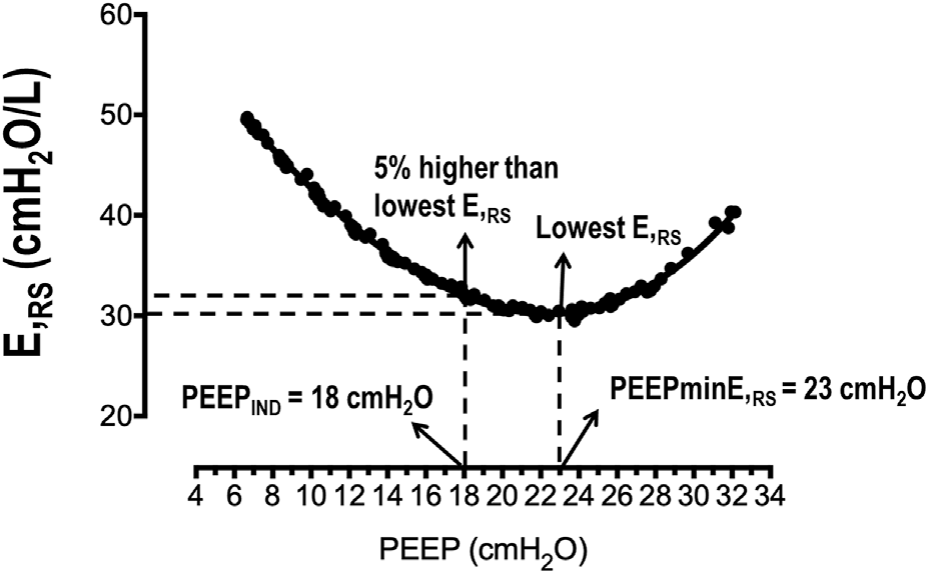
Representative PEEP–E_RS_ curve. In this patient, the PEEP associated with the lowest E_RS_ was 23 cmH_2_O (PEEPminE_RS_), and the PEEP associated with the 5% higher E_RS_ than the lowest E_RS_ was 18 cmH_2_O (PEEP_IND_). E_RS_, respiratory system elastance; PEEP, positive end-expiratory pressure.

On the day of surgery, the procedure for anesthesia and pre-surgery care, such as fasting period, was followed according to the hospital protocols (Figure 1). Briefly, patients were intubated by endo-tracheal intubation (inner diameter, 7.0 mm), according to a rapid sequence intubation protocol with intravenous administration of fentanyl 1–2 μg/kg, lidocaine 1 mg/kg, propofol 1–2 mg/kg, and succinylcholine 1 mg/kg. General anesthesia was maintained with inhaled sevoflurane in expired concentrations ≥1.4% and intravenous fentanyl 1–2 μg/kg, according to the adequacy of the depth of anesthesia. Neuromuscular blockade after tracheal intubation was maintained with intravenous administration of rocuronium 0.1–0.3 mg/kg or cisatracurium 0.02–0.05 mg/kg every 20–30 min during PNP inflation. The lungs were mechanically ventilated (Aisys, Datex-Ohmeda, Madison, WI, United States) in the 35° dorsal recumbent position using the following parameters at baseline: volume-controlled ventilation (VCV), tidal volume (V_T_) = 8 mL/kg of the predicted body weight (PBW), the respiratory rate (RR) adjusted to between 10 and 20 bpm to achieve end-tidal carbon dioxide (P_ET_CO_2_) of 35–45 mmHg, the inspiratory/expiratory (I:E) ratio of 1:2, PEEP 5 cmH_2_O, and FiO_2_ 0.5–0.6. The heart rate (HR), P_ET_CO_2_, MAP, and peripheral oxygen saturation (SpO_2_) were monitored using a multiparameter monitor (S/5; Datex-Ohmeda). After baseline, abdomen insufflation was done using a clinical CO_2_ insufflator (Baxter), which maintained the IAP at 15 mmHg (∼20 cmH_2_O) during the surgery. After clinical stabilization, the arterial blood was sampled (1 mL) at the PNP time point, and all patients underwent the initial RM, as is described later. After the initial RM, an additional RM was followed by PEEP titration to detect the PEEP level associated with the lowest E_RS_ (so-called PEEPminE_RS_). At this point, randomization and open allocation into the CTRL and PEEP_IND_ groups were performed according to a computer-generated random number list: 1) the CTRL group was ventilated with the baseline ventilator parameters; 2) the PEEP_IND_ group was ventilated with the same baseline ventilator parameters and the PEEP value associated with E_RS_ that is 5% higher than its lowest level (Figure 2). The surgery was started after both PEEP adjustments were made. No further changes to the ventilation settings were made during the surgery. At the end of surgery, the arterial blood was sampled (1 mL) again, which was still under PNP pressure (15 mmHg).

#### Initial recruitment maneuver

For the initial RM, pressure-controlled ventilation was adjusted according to the following parameters: delta inspiratory pressure, 15 cmH_2_O; RR, 10 bpm; I:E ratio, 1:1; PEEP, 5 cmH_2_O; and FiO_2_, 1. PEEP was increased to 10 cmH_2_O for 10 s, followed by an increase to 20 cm H_2_O for 10 s and to 30 cmH_2_O for 30 s. After the initial maneuver, the ventilator parameters were adjusted according to the baseline condition.

#### PEEP titration maneuver

After a similar initial RM, the ventilation mode was rapidly adjusted to VCV under a square flow curve, V_T_ of 8 mL/kg of PBW, RR of 20 bpm, I:E ratio of 1:1, and FiO_2_ = 1 and PEEP was reduced in steps of 2 cmH_2_O every 30 s, when feasible according to the MAP safe levels (>65 mmHg), starting at PEEP of 26 cmH_2_O down to PEEP of 6 cmH_2_O and ending at PEEP of 5 cmH_2_O.

#### ${\text{PEEP}}_{\text{IND}}$

The parameter chosen to individualize PEEP was E_RS_. The E_RS_ at different PEEP levels during the PEEP titration maneuver was estimated in real time by the Bdalog software written in LabVIEW version 8.2 (National Instruments, Austin, TX, United States) using the least square fitting method according to the homogeneous one-compartmental linear model as follows:

$$\text{Paw}=\text{Raw}\times \text{flow}+E_{\text{RS}}\times \text{volume}+P0,$$

where Paw is the airway pressure, Raw is the airway resistance, E_RS_ is respiratory system elastance, and P0 is the airway pressure when the flow and volume are equal to zero. A PEEP–E_RS_ curve was obtained for all patients. The PEEPminE_RS_ was recognized as the PEEP level associated with the lowest E_RS_. PEEP_IND_ represented the lowest PEEP level associated with E_RS_ that is 5% higher than the minimum E_RS_ (Figure 2).

#### Data acquisition

Airflow, Paw, HR, P_ET_CO_2_, MAP, and SpO_2_ data were collected using a multiparameter monitor. Airflow and Paw were also recorded using the serial port of the mechanical ventilator and the Bdalog software. Airflow and CO_2_ sensors were calibrated by the anesthesiologists. The time points were baseline, PNP, T1, and T2. Baseline and PNP were the time points after endotracheal intubation and during PNP, respectively. T1 and T2 were the time points at the beginning and end of surgery, respectively.

Mechanical power (MP, J/min) was calculated using the following formulas:

$${\text{MP}}_{A}=0.098\times \text{RR}\times V_{T}\times {\;(\text{Ppeak},}_{\text{RS}}\;‐\;0.5\Delta {P,}_{\text{RS}}).$$

$${\text{MP}}_{B}=0.098\times \text{RR}\times V_{T}\times \Delta {P,}_{\text{RS}}.$$

$${\text{MP}}_{C}=0.098\times V_{T}\times \text{RR}\times \left({\text{Ppeak},}_{\text{RS}}+\text{PEEP}+F/6\right)/20.$$

The formulas were obtained from previous clinical studies (Gattinoni et al., 2016; Guerin et al., 2016; Giosa et al., 2019). MP_A_ computes the static PEEP × volume, elastic, and resistive components (Gattinoni et al., 2016), whereas MP_B_ computes only the elastic component (Guerin et al., 2016). MP_C_ computes the static PEEP × volume, elastic, and resistive components with the additional benefit that inspiratory holds are not necessary (Giosa et al., 2019).

### Statistical analysis

According to previous pilot data from the respiratory physiology laboratory at the Institute of Biophysics Carlos Chagas, a sample of 10 patients per arm would provide the information (1 − β = 0.9) to identify significant (α = 0.05) differences in ΔP_RS_ as the primary outcome between CTRL and PEEP_IND_ (pilot data: CTRL, 12.0 ± 4.2 cmH_2_O vs. PEEP_IND_, 20.3 ± 4.5 cmH_2_O), taking into account an effect size d = 1.84, a two-sided test, and a sample size ratio of 1 (G*Power 3.1.9.2; University of Düsseldorf, Germany).

The primary outcome was airway driving pressure, while secondary outcomes were arterial blood gas and hemodynamic. The normality of the data was tested using the Shapiro–Wilk test. The number and relative frequency were compared by Fisher’s exact test (*p* < 0.05). The Student’s *t*-test and Mann–Whitney rank-sum test were used to compare parametric and non-parametric data, respectively. The mechanical data obtained at baseline and PNP time points were compared by paired Student’s *t*-test. Arterial blood gases, respiratory variables, and MAP obtained from the CTRL and PEEP_IND_ groups at PNP and subsequent time points were compared by two-way ANOVA followed by the Holm–Sidak multiple comparisons test (*p* < 0.05).

Pearson correlation and Bland–Altman analyses were done between the MP_A_ and MP_B_ formulas and between the MP_A_ and MP_C_ formulas to determine the levels of agreement and bias obtained. The data are expressed as means and standard deviation (SD), medians and interquartile range (IQR), or number and relative frequency (%), as appropriate. The SPSS 25 (SPSS, IBM Inc., Armonk, NY, United States) and GraphPad Prism 9.0 for MacIOS (GraphPad Software, La Jolla, CA, United States) were used. A *p* < 0.05 was considered significant.

## Results

**FIGURE 3:**
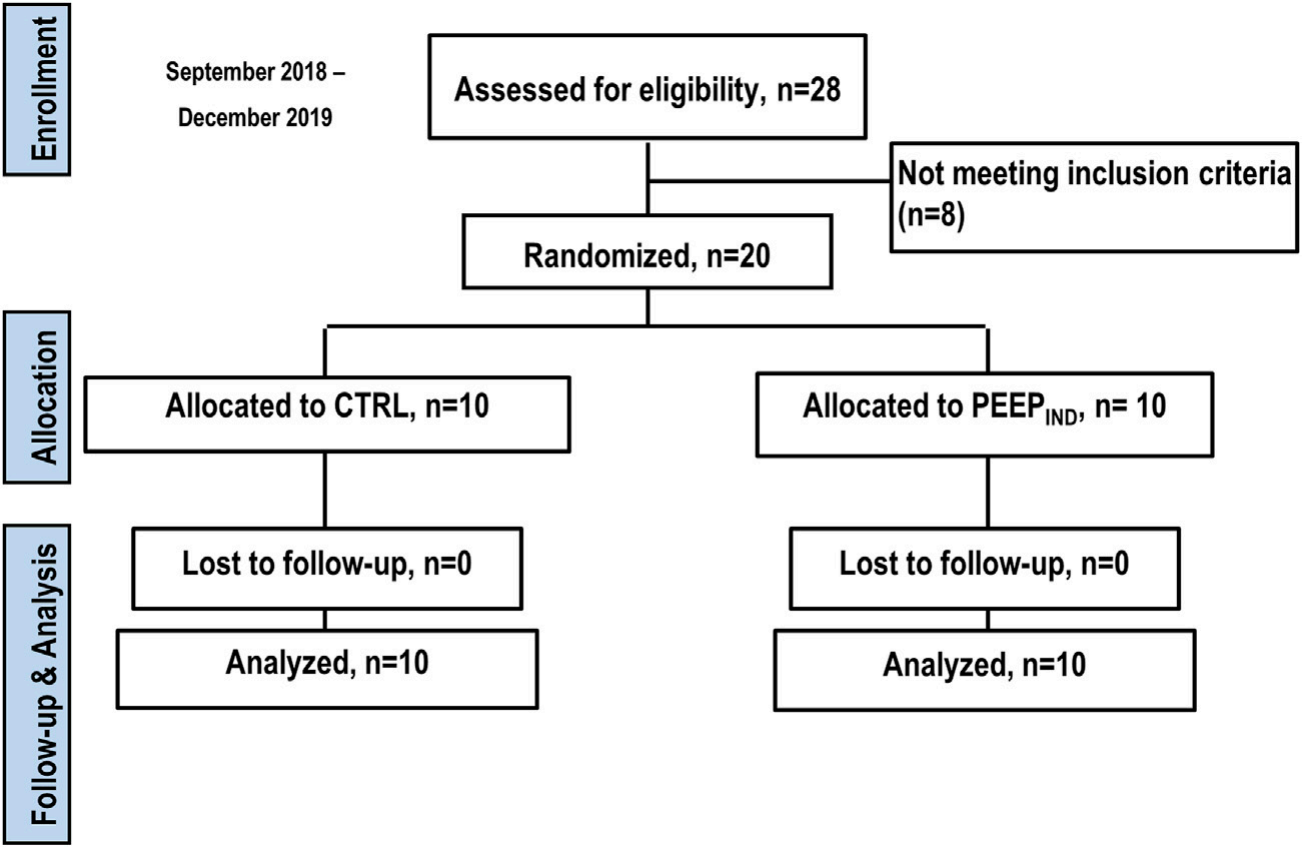
Enrolment, randomization, follow-up, and treatment. Twenty-eight patients were assessed for eligibility at the study site. Of these, 20 underwent randomization (10 to CTRL group and 10 to PEEP_IND_ group), and eight patients were excluded for not meeting the inclusion criteria.

From September 2018 to December 2019, 28 patients were assessed for eligibility at the study site. Eight patients did not meet the inclusion criteria due to chronic obstructive pulmonary disease (*n* = 4), heart disease with an ejection fraction <65% (*n* = 3), and pregnancy (*n* = 1); 20 patients underwent randomization (Figure 3). Overall, 90% of the patients were females, and the median age was 45 years (IQR, 45–52 years), the mean body mass index was 47.6 kg/m^2^ (SD, ±7.2 kg/m^2^), and most patients were American Society of Anesthesiologists physical status classification level III. Few patients were diagnosed with restrictive lung disease (15%), with 65% and 55% of them having arterial hypertension and diabetes mellitus, respectively. The surgery time was 71.5 min (IQR, 55.5–77.0 min) (Supplementary Table S1).

ΔP_RS_, MP_A_, and MP_B_ increased after the PNP procedure when compared with the baseline (*p* < 0.001, *p* = 0.004, and *p* < 0.001, respectively) (Supplementary Figure S1). Supplementary Figure S2 depicts the PEEP–E_RS_ curve for all the patients. The primary outcome ΔP_RS_ was lower in the PEEP_IND_ group at T1 and T2 than in the CTRL group (*p* < 0.001) (Table 1). PaO_2_/FiO_2_ was higher in the PEEP_IND_ group than in the CTRL group at T2 (*p* = 0.029). pHa had decreased in both groups over time (*p* = 0.002 for both). HCO_3_^−^ was lower in the PEEP_IND_ group than in the CTRL group at T2 (*p* = 0.016). No differences were observed in PaCO_2_ and lactate levels (Table 1). MAP decreased in the CTRL group at T1 and T2 in relation to the respective PNP time points (*p* = 0.011 and *p* = 0.002, respectively). In addition, MAP decreased in the PEEP_IND_ group at T2 in relation to the respective PNP time point (*p* = 0.032). No difference was observed between the PEEP_IND_ and CTRL groups at T2 (*p* = 0.445) (Table 1).

**TABLE 1:** Primary and secondary outcomes during PNP, T1, and T2.

	CTRL or PEEP_IND_	PNP	T1	T2	*p*-value: time effect	*p*-value: group effect	*p*-value: time × group effect
Primary outcome							
ΔP_RS_ (cmH_2_O)	CTRL	21 ± 3	21 ± 4	22 ± 4	<0.001	0.001	<0.001
	PEEP_IND_	20 ± 3	13 ± 1^a^ ^,^ ^b^	13 ± 2^a^ ^,^ ^b^			
Secondary outcomes							
PaO_2_/FiO_2_ (mmHg)	CTRL	236 ± 51	—	266 ± 70	<0.001	0.156	—
	PEEP_IND_	243 ± 87	—	359 ± 104*#			
pHa	CTRL	7.37 ± 0.04	—	7.32 ± 0.04	<0.001	0.736	—
	PEEP_IND_	7.37 ± 0.04	—	7.31 ± 0.04			
PaCO_2_ (mmHg)	CTRL	41 ± 4	—	41 ± 6	0.017	0.974	—
	PEEP_IND_	44 ± 4	—	44 ± 6			—
HCO_3_^−^ (mEq/L)	CTRL	24 ± 2	—	23 ± 1	<0.001	0.035	—
	PEEP_IND_	23 ± 1	—	21 ± 1*#			
Lactate (mmol/L)	CTRL	1.0 ± 0.3	—	1.2 ± 0.5	0.266	0.086	—
	PEEP_IND_	1.6 ± 0.9	—	1.6 ± 0.7			
MAP (mmHg)	CTRL	106 ± 21	91 ± 15^b^	88 ± 11^b^	<0.001	0.088	
	PEEP_IND_	92 ± 19	85 ± 7	79 ± 14^b^			
V_t_ (mL/kg PBW)	CTRL	7.8 ± 0.4	7.8 ± 0.4	7.9 ± 0.3	0.635	0.841	—
	PEEP_IND_	8.0 ± 0.7	7.8 ± 0.4	7.9 ± 0.4			
RR (bpm)	CTRL	15 ± 2	16 ± 2	17 ± 2	0.010	0.170	—
	PEEP_IND_	16 ± 2	17 ± 3	18 ± 3^b^			
Ppeak_RS_ (cmH_2_O)	CTRL	32 ± 4	31 ± 5	33 ± 4	0.004	0.183	<0.001
	PEEP_IND_	31 ± 5	36 ± 3^a^ ^,^ ^b^	36 ± 4^b^			
PEEP (cmH_2_O)	CTRL	4.7 ± 1.8	4.8 ± 1.2	4.7 ± 1.3	<0.001	<0.001	<0.001
	PEEP_IND_	4.3 ± 0.9	16.3 ± 2.5^a^ ^,^ ^b^	16.3 ± 2.5^a^ ^,^ ^b^			
E_RS_ (cmH_2_O/L)	CTRL	52 ± 10	51 ± 10	55 ± 10	<0.001	<0.001	<0.001
	PEEP_IND_	47 ± 9	31 ± 7^a^ ^,^ ^b^	32 ± 8^a^ ^,^ ^b^			
Raw (cmH_2_O/L/s)	CTRL	18 ± 6	18 ± 5	18 ± 5	0.002	0.244	0.004
	PEEP_IND_	18 ± 6	14 ± 3^b^	15 ± 4^b^			
MP_A_ (J/min)	CTRL	13 ± 3	13 ± 2	14 ± 2	<0.001	0.002	<0.001
	PEEP_IND_	14 ± 4	20 ± 5^a^ ^,^ ^b^	21 ± 5^a^ ^,^ ^b^			
MP_B_ (J/min)	CTRL	14 ± 3	14 ± 3	16 ± 3	<0.001	0.047	<0.001
	PEEP_IND_	15 ± 5	10 ± 3^a^ ^,^ ^b^	11 ± 3^a^ ^,^ ^b^			
MP_C_ (J/min)	CTRL	12 ± 2	12 ± 2	13 ± 2	<0.001	0.001	<0.001
	PEEP_IND_	12 ± 3	18 ± 4^a^ ^,^ ^b^	19 ± 4^a^ ^,^ ^b^			

Data are means ± standard deviation of 10 individuals per group. Comparisons were done by two-way ANOVA followed by Holm–Sidak multiple comparisons test (*p* < 0.05). CTRL, control group ventilated with PEEP of 5 cmH_2_O; PEEP, positive end-expiratory pressure; PEEP_IND_, PEEP adjusted at E_RS_ that is 5% higher than the PEEPminE_RS_; PNP, pneumoperitoneum; T1, at the beginning of surgery; T2, at the end of surgery; ΔP_RS_, driving pressure; PaO_2_/FiO_2_, ratio of arterial oxygen partial pressure to fractional inspired oxygen; pHa, arterial pH; PaCO_2_, carbon dioxide partial pressure; HCO_3_^−^, bicarbonate levels; MAP, mean arterial pressure; V_T_, tidal volume; PBW, predicted body weight; RR, respiratory rate; Ppeak_RS_, peak airway pressure; E_RS_, respiratory system elastance; Raw, airway resistance; MP_A_, mechanical power formula: 0.098 × RR × V_T_ × (Ppeak_RS_ − 0.5 ΔP_RS_); MP_B_, mechanical power formula: 0.098 × RR × V_T_ × ΔP_RS_); MP_C_, mechanical power formula: 0.098 × V_T_ × RR × (Ppeak_RS_ + PEEP + F/6)/20.

^a^ Versus CTRL.

^b^ Versus the respective PNP time point.

No difference was observed in V_T_ among the groups and over time (Table 1). RR increased in the PEEP_IND_ group at T2 in relation to the PNP time point (*p* = 0.019). PEEP was higher in the PEEP_IND_ group at T1 and T2 than in the CTRL group (*p* < 0.001 for both). Ppeak_RS_ was higher in the PEEP_IND_ group at T1 than in the CTRL group (*p* = 0.048). E_RS_ was lower in the PEEP_IND_ group at T1 and T2 than in the CTRL group (*p* < 0.001). Raw decreased in the PEEP_IND_ group at T1 and T2 in relation to the respective PNP time points (*p* < 0.001 for both). MP_A_ and MP_C_ were higher, whereas MP_B_ was lower in the PEEP_IND_ group than in CTRL groups at T1 and T2 (Table 1).

**FIGURE 4:**
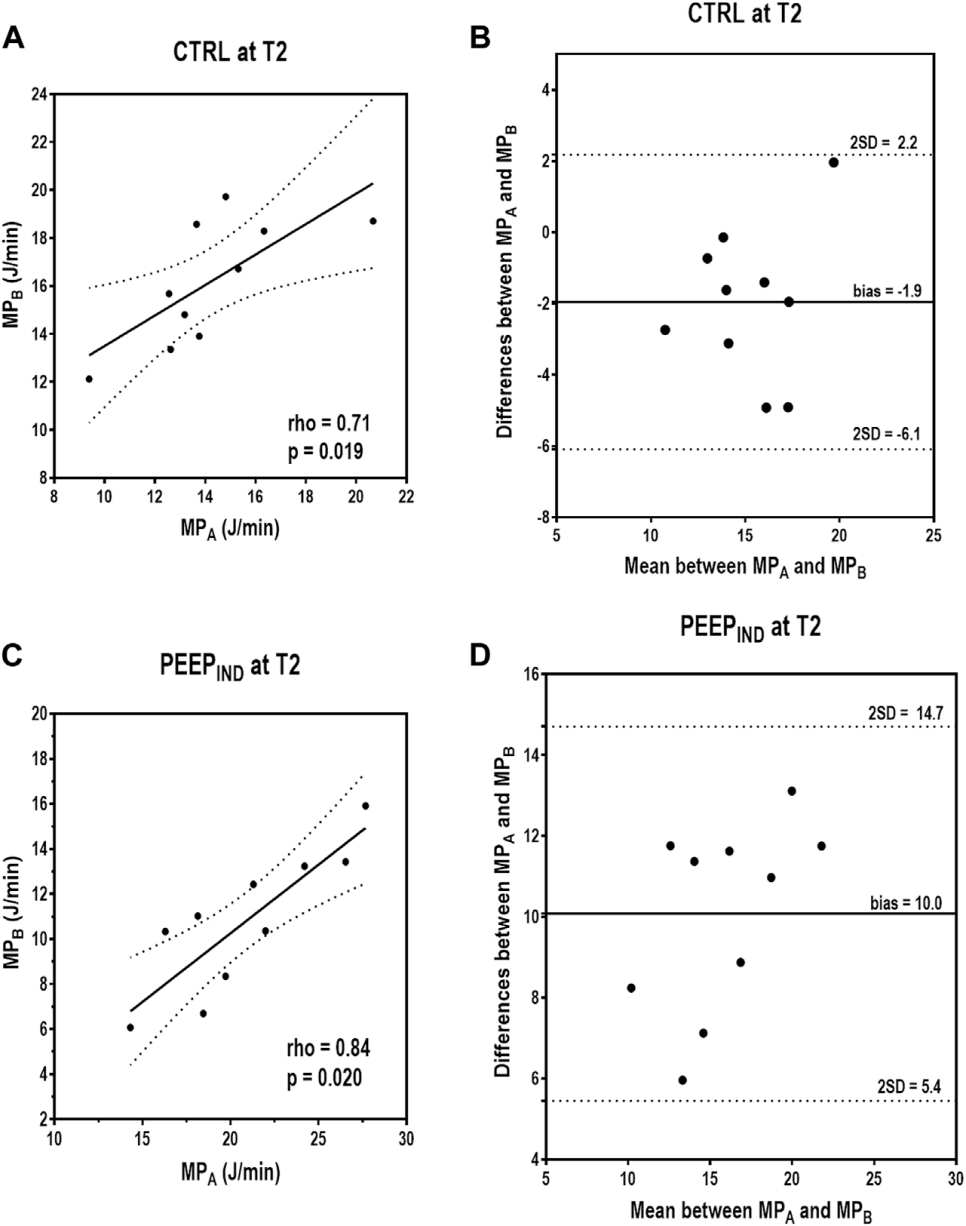
Correlations and Bland–Altman plot between MP_A_ and MP_B_ in CTRL and PEEP_IND_ groups at T2. In the CTRL group at T2, MP_A_ and MP_B_ showed a positive correlation (rho = 0.71, *p* = 0.019), and the Bland–Altman plot showed a bias of −1.9 J/min between both formulas for mechanical power **(A,B)**. In the PEEP_IND_ group at T2, MP_A_ and MP_B_ showed a positive correlation (rho = 0.84, *p* = 0.020), and the Bland–Altman plot showed a bias of +10.0 J/min between both formulas for mechanical power **(C,D)**. CTRL, control group ventilated with PEEP of 5 cmH_2_O; MP_A_, mechanical power formula: 0.098 × RR × V_T_ × (Ppeak_RS_ − 0.5 ΔP_RS_); MP_B_, mechanical power formula: 0.098 × RR × V_T_ × ΔP_RS_); PEEP, positive end-expiratory pressure; PEEP_IND_, PEEP adjusted at E_RS_ that is 5% higher than the PEEPminE_RS_; RR, respiratory rate; T2, at the end of surgery; V_T_, tidal volume.

In the CTRL group at T2, MP_A_ and MP_B_ showed a positive correlation (rho = 0.71, *p* = 0.019), whereas the Bland–Altman plot showed a bias of −1.9 J/min between both the formulas for mechanical power (Figures 4A and B). In the PEEP_IND_ group at T2, MP_A_ and MP_B_ showed a positive correlation (rho = 0.84, *p* = 0.020), while the Bland–Altman plot showed a bias of +10.0 J/min between both the formulas for MP (Figures 4C and D).

**FIGURE 5:**
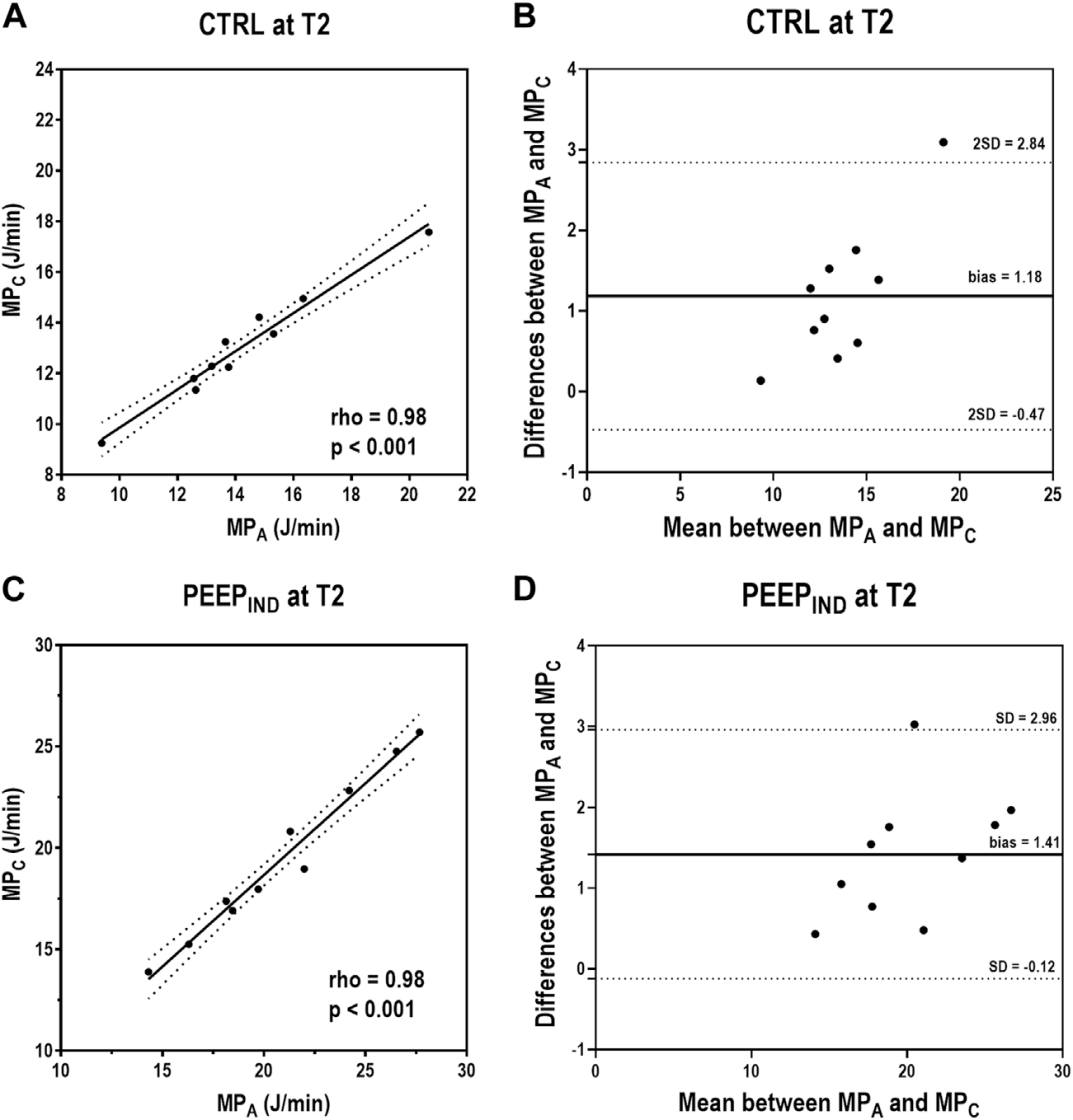
Correlations and Bland–Altman plot between MP_A_ and MP_C_ in the CTRL and PEEP_IND_ groups at T2. In the CTRL group at T2, MP_A_ and MP_C_ showed a positive correlation (rho = 0.98, *p* < 0.001), and the Bland–Altman plot showed a bias of 1.19 J/min between both formulas for MP **(A,B)**. In the PEEP_IND_ group at T2, MP_A_ and MP_C_ showed a positive correlation (rho = 0.98, *p* < 0.001), and the Bland–Altman plot showed a bias of 1.41 J/min between both formulas for MP **(C,D)**. CTRL, control group ventilated with PEEP of 5 cmH_2_O; MP_A_, mechanical power formula: 0.098 × RR × V_T_ × (Ppeak_RS_ − 0.5 ΔP_RS_); MP_C_, mechanical power formula: 0.098 × V_T_ × RR × (Ppeak_RS_ + PEEP + F/6)/20; PEEP, positive end-expiratory pressure; PEEP_IND_, PEEP adjusted at E_RS_ that is 5% higher than the PEEPminE_RS_; RR, respiratory rate; T2, at the end of surgery; V_T_, tidal volume.

**FIGURE 6:**
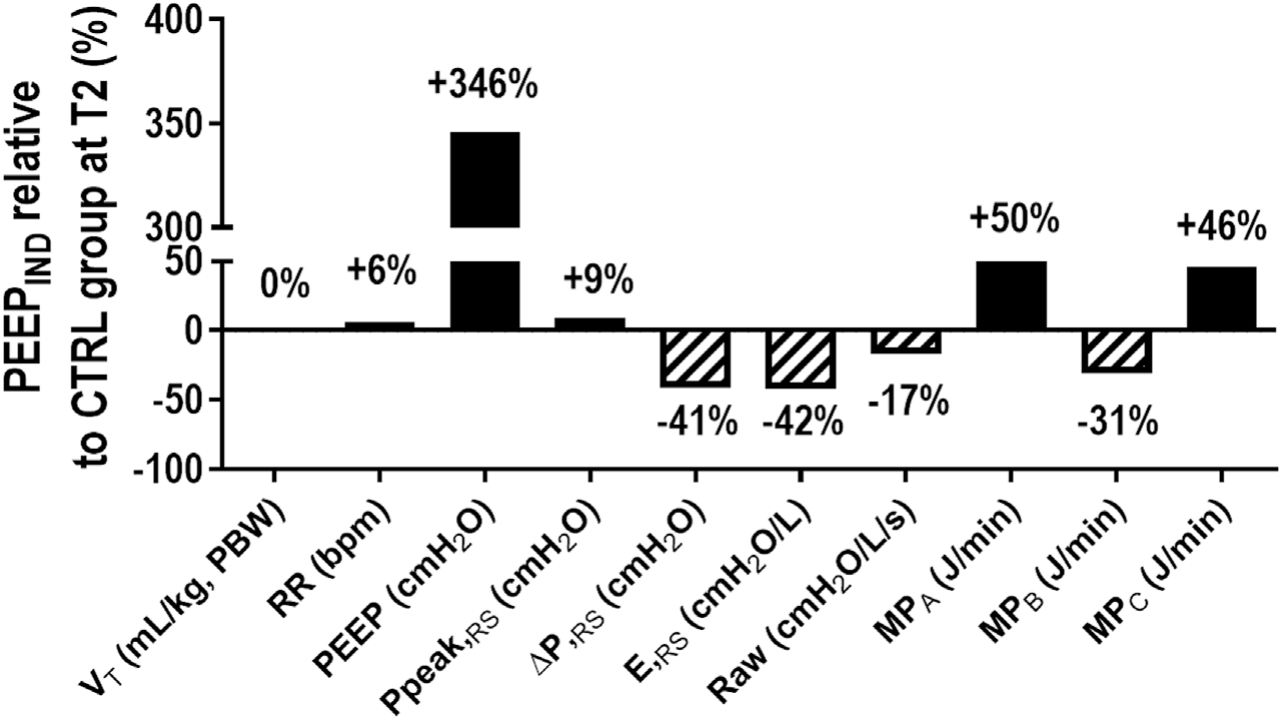
Summary of respiratory variables in the PEEP_IND_ group in relation to the CTRL group at T2. The solid bars show the increase, and the hatched bars show the decrease in percentage. The MP_A_ increase (+50%) was highly influenced by the increase in PEEP (+346%) and did not follow the decrease in ΔP_RS_ and E_RS_ (−41% and −42%, respectively). MP_B_ (−31%) decrease followed the decrease in ΔP_RS_ and E_RS_. CTRL, control group ventilated with PEEP of 5 cmH_2_O; E_RS_, respiratory system elastance; MP_A_, mechanical power formula: 0.098 × RR × V_T_ × (Ppeak_RS_ − 0.5 ΔP_RS_); MP_B_, mechanical power formula: 0.098 × RR × V_T_ × ΔP_RS_); MP_C_, mechanical power formula: 0.098 × V_T_ × RR × (Ppeak_RS_ + PEEP + F/6)/20. PEEP, positive end-expiratory pressure; PEEP_IND_, PEEP adjusted at E_RS_ that is 5% higher than the PEEPminE_RS_; Ppeak_RS_, peak airway pressure; Raw, airway resistance; RR, respiratory rate; T2, at the end of surgery; V_T_, tidal volume; ΔP_RS_, driving pressure.

In the CTRL group at T2, MP_A_ and MP_C_ showed a positive correlation (rho = 0.98, *p* < 0.001), whereas the Bland–Altman plot showed a bias of +1.18 J/min between both formulas for MP (Figures 5A and B). In the PEEP_IND_ group at T2, MP_A_ and MP_C_ showed a positive correlation (rho = 0.98, *p* < 0.001), whereas the Bland–Altman plot showed a bias of +1.41 J/min between both the formulas for MP (Figures 5C and D). Figure 6 shows a summary of the respiratory variables in the PEEP_IND_ group in relation to the CTRL group at T2 in percentage. The increase in MP_A_ (+50%) was highly influenced by the increase in PEEP (+346%) and did not follow the decrease in ΔP_RS_ and E_RS_ (−41% and −42%, respectively). The decrease in MP_B_ (−31%) followed the decrease in ΔP_RS_ and E_RS_. MP_A_ and MP_C_ showed comparative increments.

## Discussion

This single-center, randomized clinical trial on obese patients under PNP for bariatric surgery by abdominal laparoscopy showed that 1) a ventilatory strategy based on the PEEP level associated with E_RS_ that is 5% higher than the lowest E_RS_ was feasible and reduced ΔP_RS_ when compared with a CTRL group ventilated at PEEP of 5 cmH_2_O; 2) oxygenation improved in the PEEP_IND_ group compared with the CTRL group, with similar MAP; 3) the MP_A_ and MP_C_ formulas used to compute the static PEEP × volume, elastic, and resistive components did not follow the improvement observed in ΔP_RS_, whereas the MP_B_ formula, which computes only the elastic component, did.

Laparoscopic surgery involves CO_2_ insufflation into the peritoneal cavity, producing a PNP. IAP of 15 mmHg during PNP increased ΔP_RS_, likely due to the cranial shift of the diaphragm, reduction of the lung tissue could sustain the strain, and formation of atelectasis (D Antini et al., 2018). After an increase in IAP, the cardiac output may decrease with a subsequent fall in MAP (Regli et al., 2019). Although the PEEP_IND_ group showed increased levels of PEEP compared with the CTRL group, with the MAP being comparable in the two groups. Thus, we can infer that IAP was the main factor responsible for the reduction in MAP, likely due to the compression of the inferior vena cava.

RM followed by a fixed PEEP level of 12 cmH_2_O has been associated with improved oxygenation in morbidly obese patients, but it is associated with hemodynamic instability (Whalen et al., 2006). In a study on obese patients undergoing laparoscopic gastric banding (Almarakbi et al., 2009), the respiratory mechanics and oxygenation benefits were only achieved by repetitive RM followed by 10 cmH_2_O PEEP (Talab et al., 2009; Edmark et al., 2016). Nestler et al. (2017) showed that RM followed by individualized PEEP using EIT restored end-expiratory lung volume (EELV), regional ventilation distribution, and oxygenation during anesthesia. Hemodynamic instability was reported during surgery. It has been shown that PEEP levels up to 10 cmH_2_O (7.3 mmHg) cannot prevent a decline in functional residual capacity caused by grade I intra-abdominal hypertension (12–15 mmHg); they are associated with reduced oxygen delivery as a consequence of reduced cardiac output (Regli et al., 2019). To maintain EELV according to EIT or to keep E_RS_ stable, as observed in our study, PEEP levels were adjusted to 18.5 cmH_2_O and 16.3 cmH_2_O, respectively. It has been shown that targeted PEEP levels of 10, 14, and 17 cmH_2_O at IAP of 8, 12, and 15 mmHg, respectively, resulted in lower transpulmonary driving pressure (Mazzinari et al., 2020). However, PEEP was not titrated and, thus, not individualized. PEEP can counteract the negative effects of intra-abdominal hypertension on lung volume, and the present study favors setting PEEP to allow E_RS_ that is 5% higher than the minimal E_RS_.

The intraoperative ΔP_RS_, the ratio of V_T_ to respiratory system compliance, reflects the strain applied on lung tissue available for ventilation during general anesthesia (Silva and Rocco, 2018). After RM and PEEP_IND_, ΔP_RS_ decreased, likely due to an increase in lung tissue availability to accommodate V_T_. Because V_T_ did not change, we may infer that overall respiratory system compliance improved. There are currently no data from large trials regarding safe ΔP_RS_ levels for obese patients undergoing mechanical ventilation. Nevertheless, ΔP_RS_ should ideally be limited to a maximum value of 15 cmH_2_O in non-acute respiratory distress syndrome (ARDS) obese patients (Ball and Pelosi, 2019). The ΔP_RS_ observed in our PEEP_IND_ group was 13 cmH_2_O, which is within the protective range, while ΔP_RS_ was. In addition, hemodynamic instability is more frequent than respiratory impairment in obese patients without ARDS (Cinnella et al., 2013). In swine with obesity, the RM followed by PEEP titration according to the lowest E_RS_ reduced the inspiratory load on the right ventricle. A similar behavior was observed in obese patients under protective ventilation (Magder et al., 2021), and likely the protection may not only be restricted to the lungs but may also protect the heart.

MP refers to the energy transferred to the respiratory system and, similarly, to ΔP_RS_, but there are no data regarding safe MP levels for obese patients. The suggested overall protective threshold is 17–20 J/min. ΔP_RS_ and MP are dependent on chest wall elastance, which is increased in obese patients, especially during PNP (Loring et al., 2014; Ball et al., 2018). There are different formulas for MP (Gattinoni et al., 2016; Guerin et al., 2016; Giosa et al., 2019). The fundamental role of calculating MP is to inform lung protection and likely follow the behavior of key variables, such as ΔP_RS_. Here, PEEP_IND_ led to not only a reduction in ΔP_RS_ toward the protective range but also an increase in MP (>20 J/min) when the MP_A_ and MP_C_ formulas were used. The explanation relies on the contribution of the MP formula components during PEEP_IND_ ventilation. PEEP_IND_ led to a reduction in ΔP_RS_ (elastic component) by 41% when compared with the CTRL. However, to achieve that reduction, PEEP (static PEEP × volume) had to increase by 346% when compared with the CTRL and bypass the improvement observed in the elastic component (Figure 6). No major changes were observed in Raw (resistive component). On applying the MP_B_ formula, agreement with the behavior of ΔP_RS_ was observed. Thus, the choice of the MP formula may have an impact on interpreting the results, as noted by the bias in the PEEP_IND_ (+10.0 J/min) group. The MP_C_ formula has the additional benefit that inspiratory holds are not necessary, which may facilitate the inclusion of an algorithm in mechanical ventilators (Giosa et al., 2019). Because MP_C_ also computes the PEEP × volume and resistive components, it showed a similar behavior to the MP_A_ formula and did not follow the improvement observed in ΔP_RS_. The rationale for using only the dynamic elastic component or including static elastic and resistive components has been debated extensively (Huhle et al., 2018; Vasques et al., 2018). In addition, a study of 4,549 patients with ARDS showed the concept of using 4 × ΔP_RS_ + 1 × RR (4DPRR) to quantify the impact of changes in the ventilatory strategy on ventilator-induced lung injury (Costa et al., 2021). The 4DPRR formula only computes the dynamic elastic component, similar to the MP_B_ used here. Thus, we believe that our data may provide additional discussion about ventilator settings in obese patients under laparoscopy.

### Limitations

We did not compute the postoperative complications, which are highly prevalent in obese patients after laparoscopic surgeries under mechanical ventilation (Bluth et al., 2019). EIT measurements were not done and could add important information about the distribution of V_T_ and EELV in obese patients. However, we conducted the PEEP titration according to the PEEP–E_RS_ curve. We did not measure the transpulmonary pressure because there was no wide-caliber probe to keep the pylorus open, and thus, we could not pass the esophageal balloon. In addition, we did not measure invasive cardiac output and the related variables due to the absence of hemodynamic devices in the operating room.

## Conclusion

The present single-center, randomized clinical trial on obese patients under abdominal laparoscopy showed that individualized PEEP was associated with a reduction in ΔP_RS_ and improvement in oxygenation with comparable MAP. The MP, which solely computes the elastic component, better reflected the improvement in ΔP_RS_ observed in individualized PEEP group.

## Figures and Tables

**Table:** 

**ARDS**	acute respiratory distress syndrome
**ASA**	American Society of Anesthesiologists
**E**_**RS**_	respiratory system elastance
**EELV**	end-expiratory lung volume
**EIT**	electrical impedance tomography
**FEV**_**1**_	forced expiratory volume at the first second
**HR**	heart rate
**IAP**	intra-abdominal pressure
**I:E**	inspiratory/expiratory
**IQR**	interquartile range
**MAP**	mean arterial pressure
**MP**	mechanical power
**MP**_**A**_	MP that includes the static PEEP × volume, elastic, and resistive components
**MP**_**B**_	MP considering only the elastic component
**MP**_**C**_	MP that includes the static PEEP × volume, elastic, and resistive components without inspiratory holds
**PaCO**_**2**_	carbon dioxide arterial pressure
**PaO**_**2**_ **/FiO**_**2**_	oxygenation index
**Paw**	airway pressure
**PBW**	predicted body weight
**PEEP**	positive end-expiratory pressure
**PEEP**_**IND**_	individualized PEEP
**PEEPminE**_**RS**_	PEEP level associated with the lowest E_RS_
**P**_**ET**_ **CO**_**2**_	end-tidal carbon dioxide
**pHa**	arterial pH
**PNP**	pneumoperitoneum
**Ppeak**_**RS**_	airway peak pressure
**Raw**	airway resistance
**REBEC**	Brazilian Registry of Clinical Trials
**RM**	recruitment maneuver
**RR**	respiratory rate
**SD**	standard deviation
**SpO**_**2**_	peripheral oxygen saturation
**T1**	time point at the beginning of surgery
**T2**	time point at the end of surgery
**VCV**	volume-controlled ventilation
**V**_**T**_	tidal volume
**P**_**RS**_	airway driving pressure

## Data Availability

The original contributions presented in the study are included in the article/Supplementary Material; further inquiries can be directed to the corresponding author.

## References

[B1] Almarakbi W. A. Fawzi H. M. Alhashemi J. A. (2009). Effects of four intraoperative ventilatory strategies on respiratory compliance and gas exchange during laparoscopic gastric banding in obese patients. Br. J. Anaesth. 102, 862–868. 10.1093/bja/aep084 19403595

[B2] Andersson L. E. Baath M. Thorne A. Aspelin P. Odeberg-Wernerman S. (2005). Effect of carbon dioxide pneumoperitoneum on development of atelectasis during anesthesia, examined by spiral computed tomography. Anesthesiology 102, 293–299. 10.1097/00000542-200502000-00009 15681942

[B3] Ball L. Hemmes S. N. T. Serpa Neto A. Bluth T. Canet J. Hiesmayr M. (2018). Intraoperative ventilation settings and their associations with postoperative pulmonary complications in obese patients. Br. J. Anaesth. 121, 899–908. 10.1016/j.bja.2018.04.021 30236252

[B4] Ball L. Pelosi P. (2019). How I ventilate an obese patient. Crit. Care 23, 176. 10.1186/s13054-019-2466-x 31097006 PMC6524229

[B5] Bluth T. Serpa Neto A. Schultz M. J. Pelosi P. Gama de Abreu M. Wrigge H. (2019). Effect of intraoperative high positive end-expiratory pressure (PEEP) with recruitment maneuvers vs low PEEP on postoperative pulmonary complications in obese patients: a randomized clinical trial. JAMA 321, 2292–2305. 10.1001/jama.2019.7505 31157366 PMC6582260

[B6] Boberg L. Singh J. Montgomery A. Bentzer P. (2022). Environmental impact of single-use, reusable, and mixed trocar systems used for laparoscopic cholecystectomies. PLoS One 17, e0271601. 10.1371/journal.pone.0271601 35839237 PMC9286249

[B7] Boesing C. Schaefer L. Hammel M. Otto M. Blank S. Pelosi P. (2023). Individualized positive end-expiratory pressure titration strategies in superobese patients undergoing laparoscopic surgery: prospective and non-randomized crossover study. Anesthesiology 139, 249–261. 10.1097/aln.0000000000004631 37224406

[B8] Cinnella G. Grasso S. Spadaro S. Rauseo M. Mirabella L. Salatto P. (2013). Effects of recruitment maneuver and positive end-expiratory pressure on respiratory mechanics and transpulmonary pressure during laparoscopic surgery. Anesthesiology 118, 114–122. 10.1097/ALN.0b013e3182746a10 23196259

[B9] Costa E. L. V. Slutsky A. S. Brochard L. J. Brower R. Serpa-Neto A. Cavalcanti A. B. (2021). Ventilatory variables and mechanical power in patients with acute respiratory distress syndrome. Am. J. Respir. Crit. Care Med. 204, 303–311. 10.1164/rccm.202009-3467OC 33784486

[B10] D Antini D. Rauseo M. Grasso S. Mirabella L. Camporota L. Cotoia A. (2018). Physiological effects of the open lung approach during laparoscopic cholecystectomy: focus on driving pressure. Minerva Anestesiol. 84, 159–167. 10.23736/S0375-9393.17.12042-0 28679201

[B11] Edmark L. Ostberg E. Scheer H. Wallquist W. Hedenstierna G. Zetterstrom H. (2016). Preserved oxygenation in obese patients receiving protective ventilation during laparoscopic surgery: a randomized controlled study. Acta Anaesthesiol. Scand. 60, 26–35. 10.1111/aas.12588 26235391

[B12] Eichler L. Truskowska K. Dupree A. Busch P. Goetz A. E. Zollner C. (2018). Intraoperative ventilation of morbidly obese patients guided by transpulmonary pressure. Obes. Surg. 28, 122–129. 10.1007/s11695-017-2794-3 28707173

[B13] Ferrando C. Tusman G. Suarez-Sipmann F. León I. Pozo N. Carbonell J. (2018). Individualized lung recruitment maneuver guided by pulse-oximetry in anesthetized patients undergoing laparoscopy: a feasibility study. Acta Anaesthesiol. Scand. 62, 608–619. 10.1111/aas.13082 29377061

[B14] Gattinoni L. Tonetti T. Cressoni M. Cadringher P. Herrmann P. Moerer O. (2016). Ventilator-related causes of lung injury: the mechanical power. Intensive Care Med. 42, 1567–1575. 10.1007/s00134-016-4505-2 27620287

[B15] Giosa L. Busana M. Pasticci I. Bonifazi M. Macrì M. M. Romitti F. (2019). Mechanical power at a glance: a simple surrogate for volume-controlled ventilation. Intensive Care Med. Exp. 7, 61. 10.1186/s40635-019-0276-8 31773328 PMC6879677

[B16] Guerin C. Papazian L. Reignier J. Ayzac L. Loundou A. Forel J. M. (2016). Effect of driving pressure on mortality in ARDS patients during lung protective mechanical ventilation in two randomized controlled trials. Crit. Care 20, 384. 10.1186/s13054-016-1556-2 27894328 PMC5126997

[B17] Huhle R. Serpa-Neto A. Schultz M. J. Gama de Abreu M. (2018). Is mechanical power the final word on ventilator-induced lung injury?. Ann. Transl. Med. 6 (19), 394. 10.21037/atm.2018.09.65 30460268 PMC6212365

[B18] Loring S. H. Behazin N. Novero A. Novack V. Jones S. B. O'Donnell C. R. (2014). Respiratory mechanical effects of surgical pneumoperitoneum in humans. J. Appl. Physiol. 117, 1074–1079. 10.1152/japplphysiol.00552.2014 25213641 PMC4217051

[B19] Magder S. Slobod D. Assanangkornchai N. (2021). Mechanical ventilation in the obese patient: compliance, pleural pressure, and driving pressure. Am. J. Respir. Crit. Care Med. 203, 534–536. 10.1164/rccm.202009-3607ED 32997946 PMC7924569

[B20] Mazzinari G. Diaz-Cambronero O. Alonso-Inigo J. M. Garcia-Gregorio N. Ayas-Montero B. Ibañez J. L. (2020). Intraabdominal pressure targeted positive end-expiratory pressure during laparoscopic surgery: an open-label, nonrandomized, crossover, clinical trial. Anesthesiology 132, 667–677. 10.1097/ALN.0000000000003146 32011334

[B21] Nestler C. Simon P. Petroff D. Hammermüller S. Kamrath D. Wolf S. (2017). Individualized positive end-expiratory pressure in obese patients during general anaesthesia: a randomized controlled clinical trial using electrical impedance tomography. Br. J. Anaesth. 119, 1194–1205. 10.1093/bja/aex192 29045567

[B22] Regli A. Pelosi P. Malbrain M. (2019). Ventilation in patients with intra-abdominal hypertension: what every critical care physician needs to know. Ann. Intensive Care 9, 52. 10.1186/s13613-019-0522-y 31025221 PMC6484068

[B23] Schulz K. F. Altman D. G. Moher D. CONSORT Group (2010). CONSORT 2010 statement: updated guidelines for reporting parallel group randomised trials. BMJ 340, c332. 10.1136/bmj.c332 20332509 PMC2844940

[B24] Silva P. L. Rocco P. R. M. (2018). The basics of respiratory mechanics: ventilator-derived parameters. Ann. Transl. Med. 6, 376. 10.21037/atm.2018.06.06 30460250 PMC6212352

[B25] Talab H. F. Zabani I. A. Abdelrahman H. S. Bukhari W. L. Mamoun I. Ashour M. A. (2009). Intraoperative ventilatory strategies for prevention of pulmonary atelectasis in obese patients undergoing laparoscopic bariatric surgery. Anesth. Analg. 109, 1511–1516. 10.1213/ANE.0b013e3181ba7945 19843790

[B26] Vasques F. Duscio E. Pasticci I. Romitti F. Vassalli F. Quintel M. (2018). Is the mechanical power the final word on ventilator-induced lung injury?. Ann. Transl. Med. 6 (19), 395. 10.21037/atm.2018.08.17 30460269 PMC6212351

[B27] Whalen F. X. Gajic O. Thompson G. B. Kendrick M. L. Que F. L. Williams B. A. (2006). The effects of the alveolar recruitment maneuver and positive end-expiratory pressure on arterial oxygenation during laparoscopic bariatric surgery. Anesth. Analg. 102, 298–305. 10.1213/01.ane.0000183655.57275.7a 16368847

