# Supplementary material for: Individualized positive end-expiratory pressure reduces driving pressure in obese patients during laparoscopic surgery under pneumoperitoneum: a randomized clinical trial
Source: Front Physiol. 2024 Apr 5;15:1383167. doi: 10.3389/fphys.2024.1383167 (PMC11026699; doi:10.3389/fphys.2024.1383167)
Supplement: Supplementary file 1 [file DataSheet1.docx]

Supplementary Material

Supplementary Table 1. Baseline Characteristics of the Study Population

|  | **Overall (n=20)** | **CTRL (n=10)** | **PEEP_IND_ (n=10)** | **p value** |
| --- | --- | --- | --- | --- |
| Female, n (%) | 18 (90) | 10 (100) | 8 (80) | 0.473 |
| Age (years), median (25%–75% IQR) | 45 (45–52) | 48 (28–56) | 48 (19–55) | 0.950 |
| BMI (kg/m^2^), mean (SD) | 47.6 (7.2) | 46.9 (5.8) | 48.4 (8.5) | 0.631 |
| ​ASA Physical Status Classification, n (%) |  |  |  | 0.788 |
| I | 3 (15) | 2 (20) | 1 (10) |  |
| II | 7 (35) | 3 (30) | 4 (40) |  |
| III | 10 (50) | 5 (50) | 5 (50) |  |
| Restrictive lung disease, n (%) | 3 (15) | 2 (20) | 1(10) | 1.000 |
| Arterial hypertension, n (%) | 13 (65) | 5 (50) | 8 (80) | 0.349 |
| Diabetes mellitus, n (%) | 11 (55) | 4 (40) | 7 (70) | 0.369 |
| Surgery time (min), median (25%–75% IQR) | 71.5 (55.5–77.0) | 71.5 (65.5–77.5) | 72 (46–76) | 0.441 |

Data are expressed as means (standard deviation [SD]), median (interquartile range [IQR]), or number and relative frequency (%). Comparisons were done by Student’s t test (p<0.05) or Mann-Whitney test (p<0.05), according to the data distribution. The number and relative frequency were compared by Fisher's exact test (p<0.05). CTRL, control group ventilated with PEEP of 5 cmH_2_O; PEEP_IND_, PEEP adjusted at E,_RS_ 5% higher than the PEEPminE,_RS_; BMI, body mass index; ASA, American Society of Anesthesiologists.

**Supplementary Figure 1**. ΔP,_RS_, MP_A_, MP_B_ and MP_C_ increased after the PNP procedure compared with baseline. CTRL, control group ventilated with PEEP of 5 cmH_2_O; MP_A_, mechanical power formula: 0.098 × RR × V_T_ × (Ppeak,_RS_ − 0.5 ΔP,_RS_); MP_B_, mechanical power formula: 0.098 × RR × V_T_ × ΔP,_RS_); MP_C_, mechanical power formula: 0.098 × V_T_ ×RR × (Ppeak,_RS_ + PEEP + F/6)/20. PEEP, positive end-expiratory pressure; PEEP_IND_, PEEP adjusted at E,_RS_ 5% higher than the PEEPminE,_RS_; PNP, pneumoperitoneum.


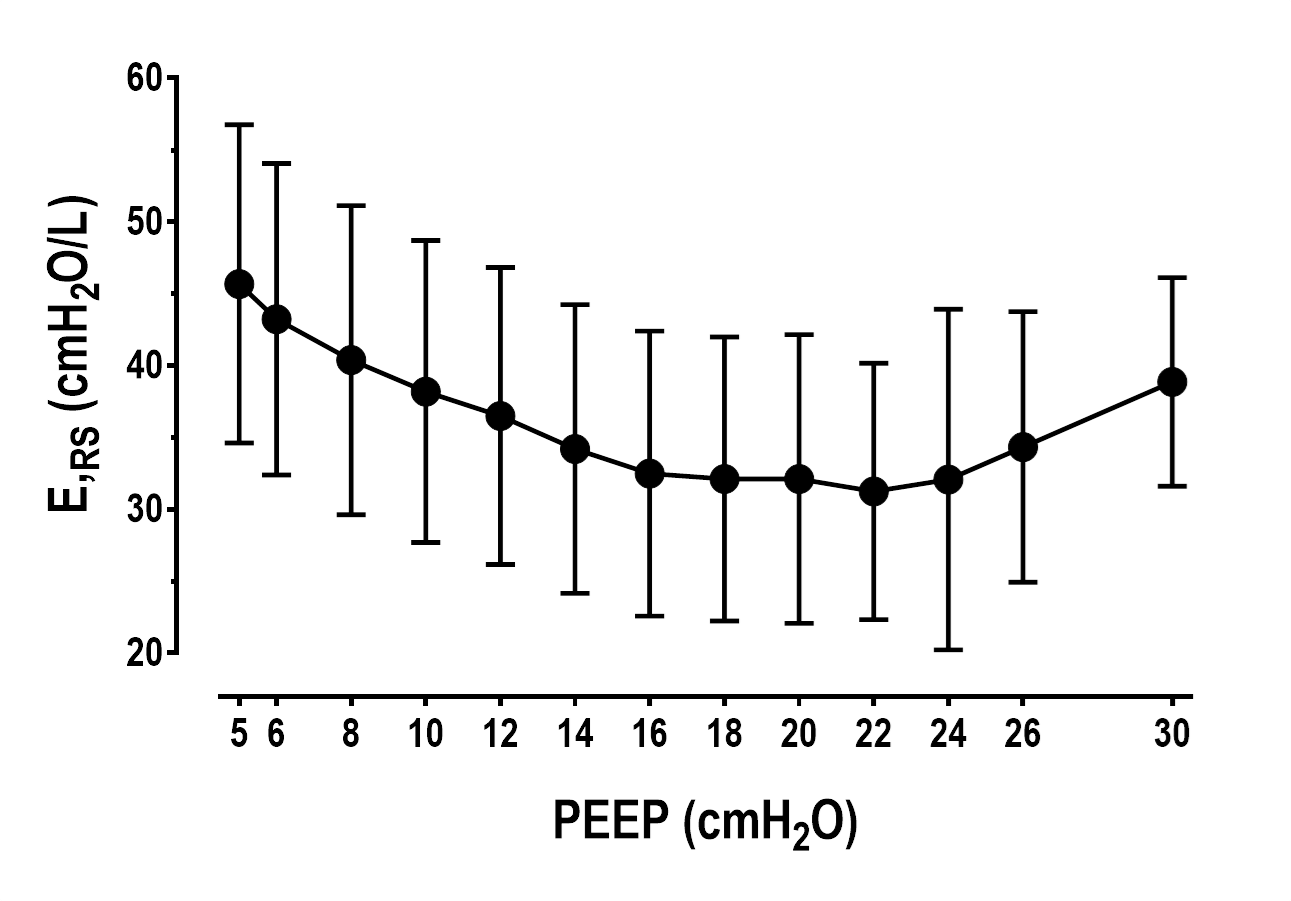


**Supplementary Figure 2**. PEEP–E,RS curve for all patients. E,_RS_, respiratory system elastance; PEEP, positive end-expiratory pressure
